# Preventing Phosphorylation of the GABA_*A*_R β3 Subunit Compromises the Behavioral Effects of Neuroactive Steroids

**DOI:** 10.3389/fnmol.2022.817996

**Published:** 2022-03-31

**Authors:** Thuy N. Vien, Michael A. Ackley, James J. Doherty, Stephen J. Moss, Paul A. Davies

**Affiliations:** ^1^Department of Neuroscience, Tufts University School of Medicine, Boston, MA, United States; ^2^Research and Non-clinical Development, Sage Therapeutics, Inc., Cambridge, MA, United States

**Keywords:** neurosteroids, GABAergic inhibition, drug-resistant seizures, anxiety, loss of consciousness

## Abstract

Neuroactive steroids (NASs) have potent anxiolytic, anticonvulsant, sedative, and hypnotic actions, that reflect in part their efficacy as GABA_*A*_R positive allosteric modulators (PAM). In addition to this, NAS exert metabotropic effects on GABAergic inhibition *via* the activation of membrane progesterone receptors (mPRs), which are G-protein coupled receptors. mPR activation enhances the phosphorylation of residues serine 408 and 409 (S408/9) in the β3 subunit of GABA_*A*_Rs, increasing their accumulation in the plasma membrane leading to a sustained increase in tonic inhibition. To explore the significance of NAS-induced phosphorylation of GABA_*A*_Rs, we used mice in which S408/9 in the β3 subunit have been mutated to alanines, mutations that prevent the metabotropic actions of NASs on GABA_*A*_R function while preserving NAS allosteric potentiation of GABAergic current. While the sedative actions of NAS were comparable to WT, their anxiolytic actions were reduced in S408/9A mice. Although the induction of hypnosis by NAS were maintained in the mutant mice the duration of the loss of righting reflex was significantly shortened. Finally, ability of NAS to terminate diazepam pharmacoresistant seizures was abolished in S408/9A mice. In conclusion, our results suggest that S408/9 in the GABA_*A*_R β3 subunit contribute to the anxiolytic and anticonvulsant efficacy of NAS, in addition to their ability to regulate the loss of righting reflex.

## Introduction

Endogenous neuroactive steroids (NAS) are synthesized in the brain from cholesterol and play a central role in regulating behavior *via* their potent anxiolytic, anticonvulsant, sedative, and hypnotic actions (Belelli et al., 2009). Accordingly, modifications in the levels of NASs contribute to anxiety, autism spectrum disorders, depression, epilepsy, neurodevelopmental disorders, and premenstrual syndrome (Belelli and Lambert, 2005; Belelli et al., 2009, 2021; Maguire, 2019; Antonoudiou et al., 2022). Significantly, NASs may also be central mediators in the efficacy of antidepressants as selective serotonin reuptake inhibitors and tricyclic antidepressants both act to increase allopregnanolone levels in the brain (Uzunova et al., 2004; Marx et al., 2006; Lüscher and Mohler, 2019). Thus, it is of fundamental importance to understand the signaling mechanism by which NASs modify neuronal excitability and the impact these processes have on behavior.

Classical mechanistic studies initially identified that NASs, in common with benzodiazepines and barbiturates, act as positive allosteric modulators (PAM) of γ-aminobutyric type A receptors (GABA_*A*_R), the principal sites of fast neuronal inhibition in the brain (Paul and Purdy, 1992; Mitchell et al., 2008). GABA_*A*_Rs are pentameric Cl^–^ selective ligand gated ion channels assembled from homologous subunits that share a common structure consisting of a large extracellular domain, four transmembrane domains (TM) with a large intracellular loop between TMs 3 and 4. GABA_*A*_R subunits can be divided into nine subunit classes, some with multiple members; α1-6, β1-3, γ1-3, δ, ε, ⊖, p, π, and ρ (Rudolph and Mohler, 2006). Consensus opinion suggests that in the brain, benzodiazepine sensitive synaptic GABA_*A*_Rs that mediate phasic inhibition are composed of α1-3, β1-3, and γ2 subunits. In contrast to this, specialized populations of extrasynaptic GABA_*A*_Rs, which are largely insensitive to benzodiazepines, but highly sensitive to NASs, are composed of α4-6, β1-3, with or without δ subunits (Bencsits et al., 1999; Mortensen and Smart, 2006; Brickley and Mody, 2012; Parakala et al., 2019).

In addition to allosteric modulation, sustained exposure to NASs can have profound effects on the expression levels of GABA_*A*_Rs, which are in part dependent upon the activity of protein kinase C (PKC; Hodge et al., 2002; Harney et al., 2003; Lambert et al., 2009). Consistent with this we have established that NASs act to increase the phosphorylation of serine 443 (S443) in the α4 subunit and serines 408 and 409 (S408/9) in the β3 subunit that are independent of their allosteric actions (Abramian et al., 2010, 2014). NAS-dependent phosphorylation promotes GABA_*A*_R insertion into the plasma membrane leading to sustained elevations in their accumulation on the cell surface. In both cultured neurons and hippocampal brain slices these events correlate with sustained increases in the efficacy of tonic inhibition (Abramian et al., 2010, 2014; Adams et al., 2015; Modgil et al., 2017). Phosphorylation of the β3 subunit can induce spontaneous channel openings of α4β3δ receptors which have an impact upon agonist/antagonist pharmacology and neuronal excitability (Wlodarczyk et al., 2013; O’Neill and Sylantyev, 2018; Dalby et al., 2020; Sexton et al., 2021).

Recently, we have demonstrated that the metabotropic actions of NASs on tonic inhibition are mediated through membrane progesterone receptor (mPR)-dependent modulation of GABA_*A*_R phosphorylation (Parakala et al., 2019). Furthermore, substitution of β3 Ser-408/9 to alanine residues (S408/9A) prevented the effects of NAS and mPR activation on tonic current while leaving the positive allosteric effects of NASs intact.

However, to date the physiological significance of this metabotropic signaling mechanism and its role in mediating the behavioral effects of NAS remains to be examined.

## Materials and Methods

### Mice

Ten- to twelve-week-old male S408/9A mice and the corresponding WT littermates were housed in a 12-h light/dark cycle with standard rodent food and water *ad libitum*. S408/9A mice (C57BL/6J background) were generated by gene targeting in murine ES cells as previously described in Vien et al. (2015). For behavioral testing, animals were acclimatized to the testing room for 1 h before the start of all procedures. Because of the confounds of studying behavioral effects of neurosteroids in female mice, only male mice were used in the current study (Martin et al., 2021). This study was carried out in accordance with the recommendations of the Institutional Animal Care and Use Committee of Tufts University and Tufts Medical Center. The protocol was approved by the Institutional Animal Care and Use Committee of Tufts University and Tufts Medical Center.

### Drug Formulation

SGE-516 [3α-Hydroxy-3β-methyl-21-(1′,2′,3′-triazol-2′-yl)-19-nor-5β-pregnan-20-one, Sage Therapeutics, Inc.] (Martinez Botella et al., 2015), and THDOC (tetrahydrodeoxycorticosterone, Sigma-Aldrich, St. Louis, MO, United States, cat. # P2016) was dissolved in vehicle (four parts saline to one part cremophor, MilliporeSigma, St. Louis, MO, United States, cat. # C5135). Diazepam (Dash Pharmaceuticals, Upper Saddle River, NJ, United States) was dissolved in vehicle. All drugs were delivered intraperitoneally, (*i.p.*) at doses specified in the text.

### Open Field Test

Individual mice were placed in the center of a 40 cm × 40 cm arena, and their movement was digitally tracked (EthoVision XT 7.0, Noldus Information Technology). WT and S408/9A mice were dosed *i.p.* with vehicle, SGE-516, or THDOC. As controls, the actions of 0.25–1 mg/kg diazepam were compared in WT and S408/9A mice. After 10 min following injections, the mice were placed in the center and the total distance traveled in the open field was compared to vehicle-treated control. Total distance traveled in 60 min was calculated.

### Light-Dark Box

Box has equally sized chambers (each chamber is 21 cm × 21 cm), individual mice *i.p.* injected with vehicle (four parts saline to one part cremophor), diazepam, SGE-516, or 10 mg/kg THDOC. After 10 min, mice were placed in the dark chamber and allowed to freely roam between the dark and light chambers. Movement and time spent in the different chambers was digitally tracked and calculated (EthoVision XT 7.0, Noldus Information Technology).

### Loss and Return of the Righting Reflex

Anesthetic sensitivity was assayed behaviorally with the loss of righting reflex according to published protocols (Jurd et al., 2003; Sun et al., 2006). Mice 11–38 weeks of age were used only once in each study and received a single injection of THDOC (100 mg/kg, *i.p*.) formulated in four parts saline to one part cremophor. As loss of righting reflex was instantaneous upon intravenous injection, mice were placed supine in a V-shaped trough with a heating pad set to 37°C to maintain body temperature during the anesthetic state. The time until return of the righting reflex was recorded by an experimenter. All studies were conducted between ZT6 and ZT8.

### Electroencephalogram Recordings and Seizures

Electroencephalograms (EEG) were performed as previously described (Vien et al., 2015). Age-matched male littermates, aged 10–12 weeks, were used for recording EEGs in awake, behaving animals. Mice were deeply anesthetized and implanted with prefabricated head mounts containing six-pin connectors with two electromyogram reference electrodes (Pinnacle Technology) 2.0 mm posterior to the bregma, along the midsagittal suture, and 2 mm below the dura. Following a 1-week recovery period, EEG activity was monitored using the Pinnacle Technology turnkey system with a 100 × amplifier and were sampled at 2 kHz and high-pass-filtered at 1 Hz (Power-Labs; AD Instruments). A 30-min baseline was obtained before an *i.p*. injection of 20 mg.kg^–1^ kainic acid (Tocris), followed by continuous EEG monitoring for an additional 120 min. Epileptiform activity was defined as electrographic events with amplitudes greater than twofold the standard deviation of the averaged baseline that last a minimum of 10 s and are separated from another event by greater than 10 s. Epileptiform activity was additionally confirmed by an increase in the power and frequency of high-amplitude events. *Status epilepticus* (SE) was defined as epileptiform activity lasting longer than 5 min with no silent period greater than 10 s. The onset of SE was defined as the time at which the gap between continuous epileptiform activity was less than 2 min. The durations of individual epileptiform events were measured from the start of the epileptiform discharge until return to baseline. Epileptiform activity was quantified using LabChart, version 7 (AD Instruments). Fast Fourier transformation was used to compare seizure power between treatments at frequencies of 1–100 Hz (Vien et al., 2015).

Sixty minutes following the onset of SE mice were given an *i.p.* injection of diazepam, THDOC, SGE-516, or the equivalent amount of vehicle consisting of four parts saline to one part cremophor. Data was converted from the time domain to the frequency domain to generate power spectral density plots [8K FFT size, Hann (cosine-bell), 87.50% window overlap]. Total power 60 s preceding drug injection were quantified and compared to the total power at 10 min after drug injection.

### Statistics

Statistical analysis for the Open Field test and Light-dark test was conducted using one-way ANOVA with *post hoc* comparison test using Holm-Sidak’s multiple comparison test. An unpaired *t*-test was used for analysis of induction and duration of loss of righting reflex, and percent change in EEG power. All analyses were done using Prism software (GraphPad).

## Results

The biochemical and electrophysiological data shown here and in previous publications (Abramian et al., 2010, 2014; Adams et al., 2015; Modgil et al., 2017; Parakala et al., 2019) collectively demonstrate that in addition to the positive allosteric modulation, NAS action at mPRs increase α4 and β3 phosphorylation leading to enhanced extrasynaptic GABA_*A*_R membrane insertion and stability resulting in increased tonic inhibition. To determine whether preventing β3 phosphorylation may lead to a modified responsiveness to the anxiolytic, hypnotic/anesthetizing and anti-convulsant properties of neurosteroids, we assessed the behavioral responses of S408/9A and WT controls to acute neurosteroid treatment.

### S408/9 Contributes to the Anxiolytic but Not Sedative Actions of Neuroactive Steroids

Neuroactive steroids are potent anxiolytics, and sedatives (Lambert et al., 2009). We have previously found that there were no significant differences between WT and S408/9A mice in motor activity and anxiety-like behavior (Vien et al., 2015). An increase in exploratory behavior in the open field test following a drug treatment is typically indicative of an increase in exploratory activity whereas a decrease in distance traveled would indicate a sedative effect of the drug used. We measured the total distance traveled in the open field in 60 min following *i.p.* doses of diazepam (DZ), SGE-516, and THDOC compared to vehicle-treated control, in WT and S408/9A mice.

**FIGURE 1:**
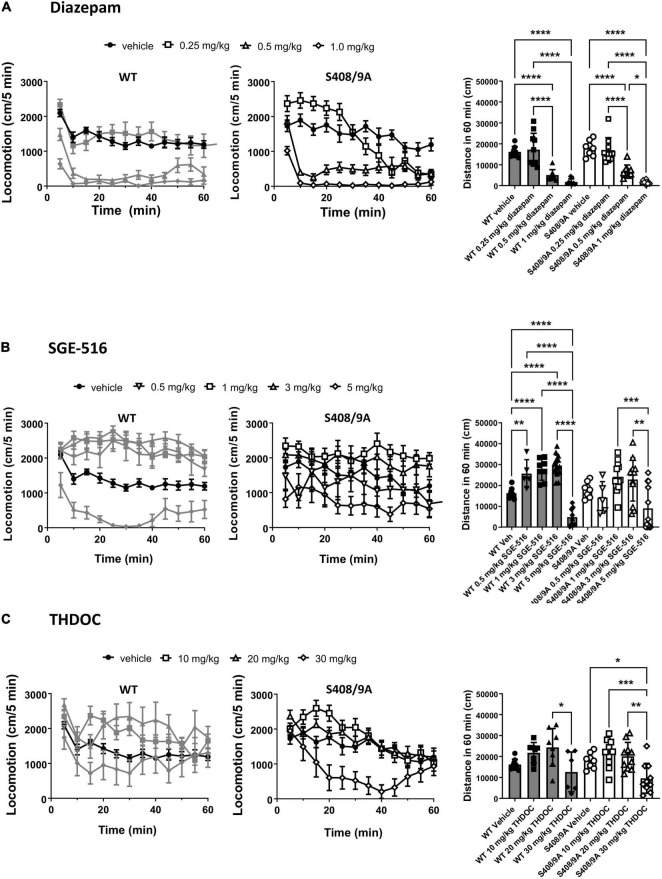
S408/9A is not critical for sedative actions of NASs in the Open Field test. WT (black bars) and S408/9A (white bars) mice were injected *i.p.* with vehicle, 0.25–1 mg/kg Diazepam **(A)**, 1–5 mg/kg SGE-516 **(B)**, or 10–30 mg/kg THDOC **(C)**. 30 min later total distance traveled was measured for 60 min. All data represent mean ± S.D., *p ≤ 0.03, **p ≤ 0.003, ***p ≤ 0.001, ****p < 0.0001.

There was no difference in distance traveled with WT vehicle treated controls (*n* = 9) compared to S408/9A vehicle controls (*p* = 0.24, *n* = 8). In both WT and S408/9A mice there were no significant change in distance traveled in mice at low dose (0.25 mg/kg, *n* = 10) of DZ compared with vehicle control (*p* = 0.6 for both WT and S408/9A, *n* = 9, Figure 1A). In WT mice SGE-516 had a significant effect on exploration at 0.5 mg/kg (*n* = 5, *p* = 0.003), 1 mg/kg (*n* = 8, *p* < 0.0001) and 3 mg/kg doses (*n* = 14, *p* < 0.0001, Figure 1B). In contrast, there was no significant increase in distance traveled following treatment with 0.5 mg/kg (*n* = 5, *p* = 0.7), 1 mg/kg (*n* = 12, *p* = 0.5), and 3 mg/kg SGE-516 (*n* = 12, *p* = 0.64, Figure 1B) in S408/9A mice. No dose of THDOC showed a significant increased travel in WT mice although there was a trend to increased travel with 10 and 20 mg/kg (*p* = 0.27, *p* = 0.09 for 10 and 20 mg/kg, respectively, *n* = 8, Figure 1C). No dose of THDOC in S408/9A mice significantly increased distance traveled (10 mg/kg, *n* = 10, *p* = 0.58: 20 mg/kg, *n* = 10, *p* = 0.75, Figure 1C).

At higher doses benzodiazepines and NASs become sedative. At 0.5 and 1 mg/kg, diazepam was strongly sedative, significantly reducing the distance traveled compared to vehicle control in WT (*n* = 8–9, *p* < 0.0001 for both 0.5 and 1 mg/kg) and S408/9A mice (*n* = 9–10, *p* < 0.0001 for both 0.5 and 1 mg/kg, Figure 1A). SGE-516 exerted sedative effects at 5 mg/kg in WT (*n* = 9, *p* < 0.0001), but in S408/9A mice, the decrease in exploration did not reach significance (*n* = 12, *p* = 0.08) compared to vehicle, but distance traveled at 5 mg/kg was significant reduced compared to 1 and 3 mg/kg SGE-616 (1 mg/kg *p* = 0.0009, *n* = 12; 3 mg/kg *p* = 0.003, *n* = 12, Figure 1B). THDOC (30 mg/kg) reduced distance traveled in WT compared to at 20 mg/kg (*n* = 6, *p* = 0.02). A significant sedative effect was observed with 30 mg/kg THDOC compared to vehicle in S408/9A mice (*n* = 10, *p* = 0.03, Figure 1C).

**FIGURE 2:**
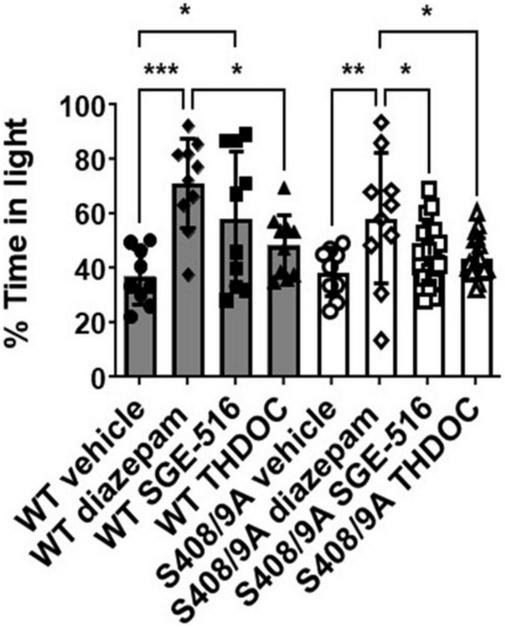
S408/9A selectively impairs the anxiolytic effects of neuroactive steroids. Time spent in the light arena of the light-dark box assay was determined for WT (black bars) and S408/9A (white bars) mice with vehicle, diazepam (0.25 mg/kg), SGE-516 (1 mg/kg), or THDOC (10 mg/kg) treatment. In both genotypes, diazepam significantly increased the amount of time spent in the light arena. THDOC was ineffective at increasing time spent in the light whereas SGE-516 increased time in light area only in WT mice. *p ≤ 0.04, **p = 0.004, ***p = 0.0006; *n* = 9–20.

To further test the anxiolytic efficacy of NASs, the behavior of both strains was examined in the light/dark box test, using non-sedating doses of diazepam (0.25 mg/kg), THDOC (10 mg/kg), and SGE-516 (1 mg/kg). For vehicle-treated mice, WT and S408/9A mice spent equal time, approximately 37% of the time, in the light (WT 221.0 ± 20.6 s, *n* = 9: S408/9A 230.5 ± 18.0 s, *n* = 9, *p* = 0.99). However, when examining the % time spent in the light 10 min following *i.p.* injection of drug there was a significant difference between genotypes (Figure 2). As expected from previous work detailing their anxiolytic actions, diazepam and the NASs increased the time in the light box. In WT mice the % time spent in the light was significantly increased with diazepam (71 ± 5%, *n* = 10, *p* = 0.0001), and SGE-516 (58 ± 8%, *n* = 10, *p* = 0.02) compared to vehicle control (37 ± 3%, *n* = 9). There was a small but insignificant increase in the % time spent in the light following THDOC treatment in WT mice (48 ± 4%, *n* = 10, *p* = 0.33).

In S408/9A mice, only diazepam significantly increased % time spent in the light (58 ± 8%, *n* = 10, *p* = 0.004) compared to vehicle control (38 ± 3%, *n* = 9). The NASs THDOC (44 ± 2%, *n* = 20, *p* = 0.67) and SGE-516 (45 ± 3%, *n* = 19, *p* = 0.47) did not significantly increase % time in the light box indicating an absence of anxiolytic effect of the NASs in S408/9A mice.

### Hypnotic Properties of Neuroactive Steroids Are Reduced in S408/9A Mice

The ability of neurosteroids to cause a loss of consciousness has long been known and one neurosteroid, alphaxalone, is used as a veterinarian general anesthetic. GABA_*A*_Rs-mediated currents are potently enhanced by the positive allosteric actions of general anesthetics (Hemmings et al., 2019). Extrasynaptic GABA_*A*_Rs are particularly sensitive to positive allosteric actions of NASs and are believed to be the primary mediators of the hypnotic endpoint of general anesthetics (Belelli and Lambert, 2005; Kretschmannova et al., 2013). Furthermore, the β3 subunit is known to be critical for hypnotic actions of general anesthetics (Jurd et al., 2003). Given that NAS activation of mPRs leads to the phosphorylation of β3 S408/9 to bring about an increase in tonic current, we hypothesized that this increase in receptor expression would, in turn, be allosterically potentiated by the NAS to produce a hypnotic effect. Given that extrasynaptic-like GABA_*A*_Rs containing the β3 S408/9A mutation are as sensitive to potentiation by THDOC as WT receptors (Adams et al., 2015) we have examined NAS-mediated loss of righting reflex (LORR) in S408/9A mice to examine the role of β3 subunit phosphorylation and receptor trafficking in NAS-mediated hypnosis.

**FIGURE 3:**
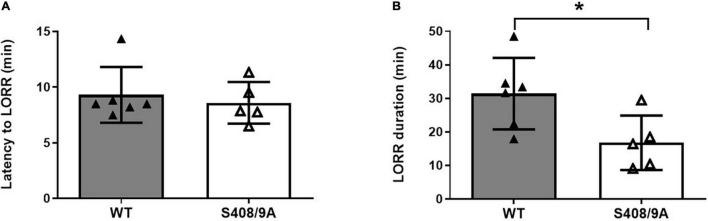
Hypnotic actions of THDOC are decreased in S408/9A mice. Hypnotic action of THDOC was measured using the loss of righting reflex assay, 44% of S408/9A mice tested did not lose their righting reflex with THDOC. Bars represent only the responders. **(A)** Induction of LORR with *i.p.* injection of 80 mg/kg THDOC was similar in WT and S408/9A mice. **(B)** The S408/9A mice spent a significantly shorter duration of loss of righting reflex compared to WT controls following THDOC (*p = 0.03; *t*-test; *n* = 5–6).

Only ∼55% of the S408/9A mice responded to 80 mg/kg *i.p.* THDOC by becoming ataxic, compared to 100% of WT controls. Of the S408/9A mice that underwent LORR, there were no statistical differences in latency of LORR compared to WT controls (WT, 9 ± 1 min, *n* = 6; S408/9A, 9 ± 1 min, *n* = 5, *p* = 0.6, Figure 3A). However, the S408/9A mice that responded to THDOC injections exhibited a striking decrease in the duration of LORR compared to WT controls (WT, 31 ± 4 min, *n* = 6: S408/9A, 17 ± 4 min, *n* = 5; *P* = 0.03; Figure 3B). This result indicates that the inability of NASs to phosphorylate β3-containing GABA_*A*_Rs reduces the hypnotic duration of NASs but not the induction.

### S408/9 Determines the Ability of Neuroactive Steroids to Terminate Pharmacoresistant Seizures

Studies in animals suggest that NASs are efficacious in terminating pharmacoresistant seizures (Rogawski et al., 2013). We explored if the ability of NASs to arrest pharmacoresistant seizures is dependent upon their ability to phosphorylate β3 subunits, by comparing the anticonvulsant efficacy of NASs in WT, and S408/9A mice using EEG recording. While these mice do not appear to exhibit overt spontaneous seizures, they are more sensitivity to kainite-induced *Status epilepticus* than WT, as reflected by a reduced latency to the onset of SE, coupled with enhanced seizure severity (Vien et al., 2015).

To examine the role that S408/9 plays in the ability of NASs to terminate pharmacoresistant seizures we implanted WT and S408/9A mice (2–4 months of age) with EEG/EMG electrodes and allowed them to recover for 7 days (Vien et al., 2015). During experiments, baseline EEGs were recorded for 30 min prior to *i.p.* injection of 20 mg/kg kainate which resulted in the development of SE approximately 35–40 min after kainate injection. Sixty min after entrance into SE, mice were treated *i.p.* with the prototypic benzodiazepine, diazepam (DZ, 20 mg/kg), THDOC (100 mg/kg), or SGE-516 (3 mg/kg). Seizure activity 10 min following treatment was compared to 30 s prior to drug treatment.

**FIGURE 4:**
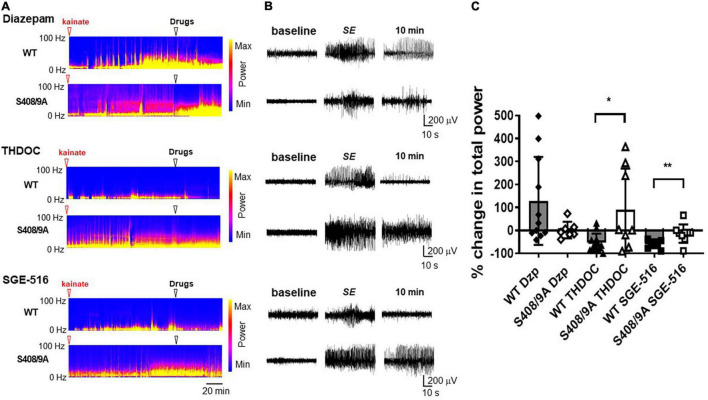
Neuroactive steroids are ineffective in terminating diazepam resistant seizures in S408/9A mice. **(A)** EEG spectrograms from WT and S408/9A mice following injection with 20 mg/kg kainate (red arrowhead) to induce *Status Epilepticus* (SE). 60 min after entrance into SE, mice were then injected *i.p*. (white arrowhead) with diazepam (Dzp, 20 mg/kg), THDOC (100 mg/kg), or SGE-516 (3 mg/kg). **(B)** EEG recordings from WT and S408/9A mice during baseline, SE, 10 min following drug administration demonstrating diazepam resistant seizures that are suppressed with THDOC or SGE-516 in WT but not S408/9A mice. **(C)** The % change in total EEG power (1–100 Hz) 10 min after drug treatment compared to 30 s prior to treatment. All data represent mean ± S.D., *p = 0.015, **p = 0.004; *n* = 7–12 mice.

Sixty minutes after entrance into SE, *i.p.* injection of DZ did not terminate seizure activity and EEG power tended to increase following DZ injection in WT mice (128 ± 58%, *n* = 11). S408/9A mice readily entered SE and DZ resistance was evident following treatment (2 ± 14%, *n* = 7; Figure 4). In WT mice, THDOC dramatically reduced seizure activity and EEG power 10 min after administration by −52 ± 39%, *n* = 12 (Figure 4). In contrast to WT mice, THDOC were unable to terminate pharmacoresistant SE in S408/9A mice (90 ± 63%, *n* = 8, *p* = 0.015, Figure 4). Similarly, the reduction in EEG power by SGE-516 treatment (−59 ± 5%, *n* = 10) in WT mice was significantly reduced in S408/9A mice (−14 ± 13%, *n* = 9, *p* = 0.004).

## Discussion

Pregnane neurosteroids such as allopregnanolone and tetrahydrodeoxycorticosterone (THDOC), and their synthetic neuroactive steroids, have long been known to have anxiolytic, sedative, hypnotic, and anticonvulsant properties (Belelli et al., 2020). Conventionally, it was thought that these pharmacological endpoints were a result of a positive allosteric modulation (PAM) of GABA_*A*_Rs, whereby neurosteroids bind to the GABA_*A*_R causing a conformational structural change to prolong channel open times and enhance Cl^–^ flow through the channel. Both synaptic and extrasynaptic GABA_*A*_Rs are sensitive to the PAM activity of neurosteroids (Belelli and Lambert, 2005). However, it has been documented that neurosteroids have long-lasting GABA_*A*_R modulatory effects, outlasting the presence of neurosteroids and thus negating any allosteric modulation. We have investigated additional mechanisms that could explain these long-term neurosteroid effects on GABA_*A*_Rs and have demonstrated that in the dentate gyrus of the hippocampus the actions of neurosteroids increase the intracellular trafficking and plasma membrane stability of extrasynaptic GABA_*A*_Rs in a phosphorylation-dependent manner (Jacob et al., 2008; Abramian et al., 2010, 2014; Comenencia-Ortiz et al., 2014). Furthermore, neurosteroids evoke this change in the phosphorylated state of α4 and β3 subunits *via* a metabotropic mechanism involving the activation of membrane Progesterone Receptors (mPRs) leading to an increase in PKC and PKA activity (Parakala et al., 2019).

Neurosteroids are known to facilitate their persistent effects, through phosphorylation-dependent mechanism (Fancsik et al., 2000; Lambert et al., 2001a,b; Brussaard and Koksma, 2003; Harney et al., 2003; Herd et al., 2007; Mitchell et al., 2008; Vithlani and Moss, 2009; Abramian et al., 2010, 2014; Kia et al., 2011; Comenencia-Ortiz et al., 2014). Therefore, an understanding of the mechanisms through which neurosteroids exert their effects on extrasynaptic GABA_*A*_Rs may promote the development of therapies to alleviate neurological disorders. In this study, we investigated the contribution of β3 subunit phosphorylation in mediating the effects of neurosteroids on GABA_*A*_R activity at the behavioral level.

Neuroactive steroids have long been demonstrated to have anxiolytic effects in several animal models of anxiety (Schule et al., 2014). Increases of NAS levels in the hippocampus are correlated with lower anxiety and increased exploratory behavior (Koonce et al., 2012). Here, we examined if this anxiolytic effect was due to allosteric or metabotropic mediated pathways. We have previously shown that β3S408/9A mice do not exhibit any deficits in locomotor activity and exhibit comparable behavior in the open field compared to WT animals (Vien et al., 2015). Similarly, we found no basal differences between WT and S408/9A mice in the open field and light-dark box. We initially examined the open field apparatus and saw an increase in exploratory behavior with SGE-516 and THDOC in WT mice that was absent in β3S408/9A mice. At high doses of THDOC and diazepam both WT and β3S408/9A mice became sedated suggesting positive allosteric modulation of GABA_*A*_Rs underlies the sedating actions of NASs. In agreement with this, sedation brought on by administration of general anesthetics is believed to be through positive allosteric modulation of GABA_*A*_Rs containing the β2 subunit (Reynolds et al., 2003).

To explore NAS anxiolytic effects, we used the light-dark box and examined the time spent in the light. In accordance with its anxiolytic properties, diazepam increased time spent in the light in both WT and β3S408/9A mice. Only SGE-516 showed anxiolytic effects in WT mice at the dose tested and no anxiolytic effect was observed in β3S408/9A mice demonstrating the requirement to activate the metabotropic pathway for NASs anxiolysis. While there was a trend for an anxiolytic effect with THDOC, it did not reach significance. Previous studies have described anxiolytic effects with THDOC (Crawley et al., 1986; Wieland et al., 1991; Rodgers and Johnson, 1998), the differences in past and present observations could be due to timing of the test following THDOC administration, dose of THDOC, different mouse strain, and different tests of anxiety employed.

A large percentage of β3S408/9A mice did not respond to the hypnotic actions of THDOC. Those mice that did eventually lose consciousness, induction of hypnosis was no different to WT mice. However, loss of righting reflex duration was significantly reduced in β3S408/9A mice. β3 subunit containing GABA_*A*_Rs are known to play an essential role in the hypnotic actions of intravenous general anesthetics (Jurd et al., 2003; Belelli et al., 2005; Belelli and Lambert, 2005; Ferguson et al., 2007). Additionally, phosphorylation of the β3 subunit is important for propofol mediated hypnosis (Nikaido et al., 2017) leading to the suggestion that modulation of phosphorylation and membrane trafficking of GABA_*A*_Rs could be targeted to enhance hypnotic endpoint of general anesthetics and reduce the dose of anesthetic needed for hypnosis and reduce their unwanted side effects.

*Status Epilepticus* (SE) in humans and rodent chemical-convulsant models, progressively becomes insensitive to arrest by benzodiazepines, the current standard of care. Development of benzodiazepine pharmacoresistance leads to a dramatic increase in mortality and morbidity (DeLorenzo et al., 1995; Lowenstein and Alldredge, 1998; Jones et al., 2002). Pharmacoresistant epilepsy accounts for approximately one third of all epileptic patients (Laxer et al., 2014). Not only have neuroactive steroids been shown to be anticonvulsant but ALLO and SGE-516 have been demonstrated to be effective at stopping pharmacoresistant epilepsy in refractory SE models (Rogawski et al., 2013; Althaus et al., 2017; Hammond et al., 2017). Both THDOC and SGE-516 terminated diazepam resistant SE in WT mice but these NASs were ineffective in β3S408/9A mice. Because NAS are still capable of acting as a PAM of tonic current in β3S408/9A mice (Adams et al., 2015), the allosteric actions of NASs which have typically been used to explain their anticonvulsant properties in pharmacoresistant epilepsy are at odds with our observations. While the S408/9A mice have increased sensitivity to kainite induced seizures (Vien et al., 2015), our results demonstrate that S408/9 or their phosphorylation status contribute to the efficacy of NAS to terminate pharmaco-resistant SE.

Great effort has been devoted to the development of GABA_*A*_Rs PAMs with the aim of producing more efficacious and potent anxiolytic, hypnotic and anticonvulsant therapies. Recognition of the metabotropic pathways whereby NASs can increase the trafficking of GABA_*A*_Rs and enhance neuronal inhibition without allosterically causing sedation will allow novel therapies to be developed. The S408/9A mutation altered the anxiolytic, hypnotic/anesthetizing, and anticonvulsant response to acute neurosteroid treatment while leaving the sedative response in S408/9A mice unaltered. We propose that the mechanism that determines neurosteroid-mediated anxiolysis, duration of LORR, and termination of pharmaco-resistant seizures involves β3 S408/9 phosphorylation and the subsequent trafficking and stabilization of extrasynaptic GABA_*A*_Rs.

## Data Availability Statement

The original contributions presented in the study are included in the article/supplementary material, further inquiries can be directed to the corresponding authors.

## Ethics Statement

The animal study was reviewed and approved by the Institutional Animal Care and Use Committee of Tufts University and Tufts Medical Center.

## Author Contributions

TV, MA, JD, SM, and PD contributed to the conception and design of the study. TV and PD acquired, analyzed, and interpreted the data and wrote the first draft of the manuscript. All authors contributed to manuscript revision, read, and approved the submitted version.

## Conflict of Interest

MA and JD were employed by Sage Therapeutics, Inc. SM serves as a consultant for Sage Therapeutics, Inc., and AstraZeneca, relationships that are regulated by Tufts University. The remaining authors declare that the research was conducted in the absence of any commercial or financial relationships that could be construed as a potential conflict of interest.

## Publisher’s Note

All claims expressed in this article are solely those of the authors and do not necessarily represent those of their affiliated organizations, or those of the publisher, the editors and the reviewers. Any product that may be evaluated in this article, or claim that may be made by its manufacturer, is not guaranteed or endorsed by the publisher.
